# Exploring the Patient’s Lifeworld: A Qualitative Study of Personalizing Language in Electronic Health Records

**DOI:** 10.1007/s11606-026-10396-5

**Published:** 2026-03-31

**Authors:** Akanksha Suresh, Priyanka Fernandes, Ayah Zirikly, Elaine C. Thompson, Anne R. Links, Keith Harrigian, Brant Chee, Mark Dredze, Mary Catherine Beach, Somnath Saha

**Affiliations:** 1 School of Medicine, Johns Hopkins University, Baltimore, MD USA; 2 Department of Computer Science, Whiting School of Engineering, Johns Hopkins University, Baltimore, MD USA; 3 Department of Computer Science, District of Columbia, George Washington University, Washington, USA; 4 Department of Otolaryngology-Head and Neck Surgery, Johns Hopkins School of Medicine, Baltimore, MD USA; 5 Department of Medicine, School of Medicine, Johns Hopkins University, Baltimore, MD USA; 6 Applied Physics Lab, Johns Hopkins University, Baltimore, MD USA; 7 Center for Health Equity, Johns Hopkins University, Baltimore, MD USA; 8 Center for Humanizing Medicine, Johns Hopkins University School of Medicine, Baltimore, MD USA; 9 Berman Institute of Bioethics, Johns Hopkins University, Baltimore, MD USA

**Keywords:** personalizing, stigma, bias, language, electronic health record

## Abstract

**Background:**

Physicians’ understanding of patients as persons can bolster relationships and help patients feel seen. Including personal details in electronic health records (EHR) may enhance care, but the types of details physicians document about patients’ lifeworlds remain largely uncharacterized.

**Objective:**

To assess the prevalence and types of personalizing language written by physicians in clinical encounter notes.

**Design:**

Cross-sectional qualitative content analysis of physician-written history and physical (H&P) notes.

**Patients:**

Adult patients admitted to the internal medicine inpatient service at an urban academic tertiary care hospital.

**Approach:**

We performed an inductive content analysis of H&P notes. Two authors independently coded instances of personalizing language: details about patients’ unique identities, social context, life experiences, and perspectives. The study team met periodically to discuss examples and categories and compare assessments until thematic saturation was reached. We summarized the prevalence of personalizing language and described its content.

**Key Results:**

We reviewed 570 H&P notes written about 437 unique patients (median age 57; 53% Black, 38% White; 54% male) by 186 physicians. More than half of notes (60%) contained at least one instance of personalizing language (median 2, range 1–7 per note). We identified 840 instances of personalizing language across seven domains: family (41%), work/education (32%), residence (21%), personal interests (2.9%), pets (1.8%), travel (0.6%), and goals/priorities (0.6%). Nearly half (47%) referred to general social context; 51% provided more detailed information. Only 1.9% described patients’ thoughts, feelings, or goals. Personalizing language was more commonly used in notes for men vs women (70% vs 60%, p = 0.02).

**Conclusions:**

Most H&P notes included personalizing language, though many provided limited detail and very few described patients’ interests, goals, and priorities. Future studies should evaluate patient perceptions of personalizing language in the medical record and its impact on physician attitudes towards and relationships with patients.

## INTRODUCTION

Person-centered care calls for recognizing a patient as not merely a body with a disease or recipient of medical interventions, but rather as a person with unique identities, social contexts, life experiences, and perspectives outside of healthcare. Treating patients as individuals and prioritizing their psychological, emotional and value-based needs alongside their medical care strengthens the therapeutic relationship between patients and their providers,^1,2^ allows patients to feel seen as their whole person,^3,4^ and has strong potential to alleviate stereotyping and bias.^5,6^

One powerful example of personalization within healthcare is the “My Life, My Story” program, a narrative medicine initiative in the Veterans Affairs healthcare system.^2,7^ In this program, patients work collaboratively with writers to craft a one-page narrative of their lives that becomes integrated into their electronic health record (EHR). The program has been well received by both patients and clinicians. Patients appreciate being seen in the context of their lives and feel their clinicians understand them better, and clinicians and trainees report stronger empathy and better understanding of patients’ contexts.^2,7^ The positive impact of the “My Life, My Story” program suggests that if clinicians routinely included personalizing details about patients in their notes – details that would live within the EHR and be accessible to other clinicians – they could promote person-centered care by humanizing each patient, helping them to be better known as a person and avoid being reduced to a diagnosis.^8^ They may also help patients who read their own notes feel more known and valued as persons.^9^

EHR notes, however, are written primarily to communicate clinical information to other clinicians and care team members and/or to facilitate coding and billing. As such, many notes are highly structured, problem-focused, and copied forward, which can improve efficiency but also contribute to long, data-heavy notes that prioritize biomedical and deprioritize personalizing information.^10^ Moreover, unlike the patient-driven narratives of “My Life, My Story,” personalizing language written by clinicians is filtered through the clinician’s perspectives and may or may not represent the types of personal details likely to humanize patients. In this context, patients reading their own notes may struggle to read or recognize personal details about themselves,^11^ or to perceive them as reflecting their own identities or experiences. Thus, while personalizing language describing patients’ identities, social contexts, and life experiences holds promise as as a way of representing patients’ lifeworlds in routine notes, the degree to which such language accomplishes these goals remains unclear.

To our knowledge, no studies have investigated the extent and types of personalizing details that are included in clinical notes. We therefore analyzed clinical notes written by physicians to describe the prevalence and types of personalizing language found in routine EHR documentation.

## METHODS

### Study Design and Sample

We qualitatively reviewed History and Physical (H&P) notes written from January to December 2017 by physicians in an inpatient internal medicine setting at an urban, academic, tertiary care hospital. The inpatient setting was selected because it presents a unique context in which patients often experience a loss of personal identity and autonomy due to the structure of care delivery,^12–14^ making it a particularly relevant setting for examining how personalizing language may promote person-centered care.

Notes were selected via random sampling from all encounters in which patients were admitted to an inpatient internal medicine service. For each eligible hospital encounter, all associated notes coded in the EHR metadata as “History and Physical” were considered. We focused on H&P notes because these notes, typically written at the time of hospital admission, tend to be the most comprehensive in terms of documenting patients’ clinical and social histories and often serve as a reference for care team members and consultants seeking details about the patient. Patients may have had more than one H&P note within a given hospitalization if, for instance, they were transferred between hospital units. Notes were included regardless of the source of patient history (e.g., patient, family member, other sources).

All notes were stored and accessed in a secure and encrypted virtual environment that could only be accessed by study team members. Abstracted data included basic patient demographics (age, race, ethnicity, and gender) – derived from structured fields in the EHR – and physician-author role (attending, fellow, or resident) and demographics (race, ethnicity, and gender) – derived from Human Resources Department files. The Johns Hopkins University institutional review board approved the study (IRB00363939). This study followed the Standards for Reporting Qualitative Research reporting guideline.^15^

### Data Analysis

#### Qualitative Analysis

We performed a content analysis of the entire text of H&P notes, from the history of present illness through the assessment and plan, encompassing both templated and unstructured free-text sections. We defined “personalizing language” as any text within the note that provided information about the patient’s lifeworld including their unique identities, social contexts, life experiences, or perspectives outside of their immediate medical history or presenting illness.^16^ Content analysis as a qualitative method explores the “characteristics of language as communication with attention to the content or contextual meaning of the text,” and allows for the “interpretation of the content of text data through the systematic classification process of coding and identifying themes or patterns.”^17^ We used an inductive approach, without preconceived categories, because our aim was descriptive: to systematically identify and categorize the types and frequency of personalizing language in inpatient H&P notes. This approach allowed us to identify both manifest content (explicit personal details, such as family, work, or residence) and latent content (how these details conveyed aspects of patients’ lifeworlds), aligning with our objective of characterizing how physicians document patients as persons.

Two authors (A.S. and P.K.F.) independently coded all notes, with each serving as primary coder for approximately half the sample and secondary coder for the remaining half. Any discrepancies were resolved through discussion that resulted in abstractions being retained, split into multiple abstractions or combined, or recategorized. The two authors first read 100 notes and coded instances of personalizing language about the patient, as defined above. An instance was defined as a span of text that expressed a single, complete thought, analogous to an utterance in spoken language.^18^ We focused on language describing a patient’s lifeworld and did not document personal details related to a patient’s medical history (e.g., family history of disease) or current symptoms. For example, we omitted descriptions of recent travel when mentioned solely to assess illness risk.

After reviewing 100 notes, the study team met to discuss examples and emerging themes of personalizing language. Using these themes and team insights, the two primary coders then reviewed three additional sets of 100 notes, meeting periodically together and with the study team to compare assessments, discuss ambiguous text, and redefine theme definitions. After four rounds of reviewing 100 notes (400 notes total), the team reached consensus on the major themes of personalizing language. The coders then reviewed one additional set of 170 notes without further themes emerging, suggesting thematic saturation. Lastly, we classified each instance by depth of personalization (general, specific, or referencing the “inner lifeworld”) to capture how much individualized information was conveyed.

To protect patient confidentiality, all note excerpts included in this paper were edited to modify or generalize potentially identifying details (including names, ages, rare conditions, and specific locations) while preserving the original meaning and illustrative intent.

#### Quantitative Analysis

For all included H&P notes, we first identified the number of unique patients and summarized their demographic characteristics (age, race, ethnicity, and gender), as well as the number of unique physician-authors and their roles (attending, fellow, resident) and demographics (race, ethnicity, and gender). We then calculated the proportion of notes that contained at least one instance of personalizing language, and the proportions of patient subgroups who were described with – and physician subgroups who used – personalizing language. For each content category of personalizing language, we summarized the total count and percentage of instances, and within each category, determined the count and percentage of instances classified as general, specific, or references to patients’ “inner” lifeworld, i.e., references describing patients’ thoughts, feelings, goals, values, priorities, etc. We examined differences in proportions of notes containing personalizing language by patient and physician characteristics, using Pearson’s chi-square tests or Fisher’s exact tests.

## RESULTS

### Study Sample

The 570 reviewed H&P notes came from 438 unique hospitalizations, indicating that there were 132 instances of multiple H&P notes within a single hospitalization. Notes in our sample were written by 193 physicians about 437 unique patients, indicating that there was one patient in our random sample with 2 hospitalizations. Of the 570 notes, the majority were internal medicine ward team notes (*n* = 472, 83%), 49 (8.6%) were intensive care unit notes, and 29 (5.1%) were pre-procedure notes. The remainder (*n* = 20) included primarily H&P notes from consulting services or the emergency department.

About half (*n* = 233, 53%) of patients were identified as Black, 38% as White (*n* = 167), and 8% (*n* = 37) as another race; 4% (*n* = 16) were identified as Hispanic. Over half (*n* = 237, 54%) were identified as male; median age was 57 (range 19–93) (Table 1). Physicians included attendings (*n* = 46, 24%), fellows (*n* = 50, 26%), and residents (*n* = 97, 50%). Most were White (*n* = 114, 59%), non-Hispanic (*n* = 188, 97%), and male (*n* = 122, 63%) (Table 2).

**Table 1:** Use of Personalizing Language by Patient Characteristics

Patients (*N* = 437)	Personalizing Language Used	No Personalizing Language Used
**Race**	**n (%)**	**n (%)**
Black (*n* = 233)	160 (69)	73 (31)
White (*n* = 167)	106 (63)	61 (37)
Other (*n* = 37)	21 (57)	16 (43)
	*p* = 0.27	
**Ethnicity**	**n (%)**	**n (%)**
Hispanic or Latino (*n* = 16)	9 (56)	7 (44)
Not Hispanic or Latino (*n* = 421)	278 (66)	143 (34)
	*p* = 0.42	
**Age**	**n (%)**	**n (%)**
19–39 (*n* = 88)	60 (68)	28 (32)
40–59 (*n* = 161)	99 (61)	62 (39)
60–79 (*n* = 157)	106 (68)	51 (32)
80–99 (*n* = 31)	22 (71)	9 (29)
	*p* = 0.55	
**Gender**	**n (%)**	**n (%)**
Female (*n* = 200)	120 (60)	80 (40)
Male (*n* = 237)	167 (70)	70 (30)
	*p* = 0.02	

Row percentages are shown for each patient characteristic

p-values from Pearson chi-square tests

**Table 2:** Use of Personalizing Language by Physician Characteristics

**Physicians (*n* = 193)**	**Personalizing Language Used**	**No Personalizing Language Used**
**Role**	**n (%)**	**n (%)**
Faculty (*n* = 46)	29 (63)	17 (37)
Fellow (*n* = 50)	28 (56)	22 (44)
Resident (*n* = 97)	80 (82)	17 (18)
	*p* <.001*	
**Race**	**n (%)**	**n (%)**
White (*n* = 114)	82 (72)	32 (28)
Asian (*n* = 59)	42 (71)	17 (29)
Black (*n* = 13)	9 (69)	4 (31)
Other/Unknown (*n* = 7)	4 (57)	3 (43)
	*p* = 0.94*	
**Ethnicity**	**n (%)**	**n (%)**
Hispanic or Latino (*n* = 5)	5 (100)	0 (0)
Not Hispanic or Latino (*n* = 188)	132 (70)	56 (30)
	*p* = 0.32**	
**Gender**	**n (%)**	**n (%)**
Female (*n* = 71)	50 (70)	21 (30)
Male (*n* = 122)	87 (71)	35 (29)
	*p* = 0.88*	

Row percentages are shown for each physician characteristic

^*^p-values from Pearson’s chi-square tests

^**^p-value from Fisher’s exact test

The review process yielded 845 initial abstractions of personalizing language; coders agreed on 805 (95%) and discussed 40 (5%) discrepancies. Of the 40 discussed, 5 were excluded, while the remaining abstractions were retained, split, combined, or recategorized by consensus.

### Prevalence of Personalizing Language

Across 570 notes, 343 (60%) contained at least one instance of personalizing language, with a range of 1–7 instances within those notes.

Table 1 summarizes the proportions of patients described with personalizing language across key demographic characteristics. We found no significant differences in the use of personalizing language by patient race, ethnicity, or age group. However, a smaller proportion of female compared to male patients were described with personalizing language (60% vs 70%, p = 0.02).

Table 2 shows the proportions of physicians that wrote personalizing language across physician subgroups and demographics. More residents compared to fellows and faculty (82% vs 56% vs 63% of faculty, *p* < 0.001) used personalizing language at least once, with similar use across physician race, gender, and ethnicity.

### Types of Personalizing Language

The 840 instances of personalizing language fell into one of seven content areas: family/important relationships, work/education, residence/living situation, personal interests, pets, travel, and goals/priorities. Within each of those content areas, we found different degrees of personalization (Table 3): nearly half of the instances (47%) referred to general aspects of social context, usually in a cursory way, 51% provided more granular information about life circumstances, and 1.9% described patients’ “inner” lifeworld.

**Table 3:** Sub-classification of Personalizing Language

**Topic**	References to Social Context		References to Inner Lifeworld
	General	Specific	
Family*	Mention of family members	Family member names, ages, roles	Significance/meaning of relationships
Work/education	General area of work/study, employment status	Specific role or details about workplace	Significance/meaning of work
Residence/places	General mention of where pt lives	Specific locations or descriptions of residence	Preferences of where pt wants to live
Interests	General mention of hobbies	Details about personal interests	Significance/meaning of activities
Pets	Mention of having a dog, cat, etc	Names or breeds	Importance of pet to patient
Travel	General mention of recent travel	Specific locations	Significance/meaning of places/vacations
Goals/priorities		Allusion to life goal/priority through a specific example	Life goals/priorities

^*^Includes friends, caregivers

#### Family/Important Relationships

Physicians recorded a wide range of information about their patients' family dynamics (*n* = 342, 41% of all instances), ranging from general references to more personal insights. The most common (67%, *n* = 230) entries were general references to family members, without specific details or personal context. Examples include "*stays with daughter and watches grandchildren*," and "*Husband in the military*."

A smaller proportion of entries (29%, *n* = 100) included specific details such as names, ages, or roles within the family. For example, one note included, "*Fiancé is in the army but visits on weekends,*” while another stated, “*Grew up in a foster family, moved back to his family a couple years ago*." Few instances (4%, *n* = 12) described more meaningful aspects of relationships, such as grief and emotional significance, e.g., "*He notes his wife recently died of liver failure, and he wishes that he had died instead of her, saying “it should have been me.”"*

#### Work/Education

Physicians recorded a variety of career and educational details (*n* = 269, 32% of all instances). Some career-related entries (13%, *n* = 36) provided general references to the patient’s field of work or area of study. Examples include "*She used to work at an ALF*" and "*Bachelors.*" These entries gave a broad sense of the patient’s career or education but did not include personal achievements or roles within the workplace. The majority of entries (85%, *n* = 229), however, included descriptions of the patient’s specific job roles, university or major, or notable career milestones. Examples include, "*worked as a journalist for over a decade*," “*Attended Cornell,”* "*he is a clergyman and has been actively involved in missionary work/pastoring for over 20 years*," and "*Served in Korea and worked in town at the family grocery store*."

A smaller number of entries (1%, *n* = 4) focused on the personal significance of work or education. For example, one stated, "*Mr. […] is a retired Army nurse, he previously worked in the ICU, orthopedics and burns. He would like to return to work as a nurse, but he has been unable to*," which not only described the patient’s job but also ascribed personal context and significance to it.

#### Residence/Living Situation

Physicians recorded information regarding residence (*n* = 180, 21% of all instances), capturing current and past living situations that ranged from general geographic mentions to more detailed accounts of living conditions. Within this category, the most common entries (63%, *n* = 114) simply noted the place of residence, often without much detail. Examples include statements like "*Lives in Bowie*," "*Patient is currently homeless,*" and “*Lives alone.”* These references provided a broad sense of the patient’s geographic context or housing circumstance but did not include specific characteristics of the residence or details about the living situation.

A smaller proportion of entries (37%, *n* = 66) included more detailed descriptions of the patient’s residence, offering context about the type of housing or the geographic location. For example, "*Lives in 100 year old rowhome*," and “*Lives by himself in a 2 story carriage house*" illustrate where the patient resides and their living conditions. Other examples include "*He lives in Owens Mills. Pt is originally from Delaware*," "*She is a refugee from the Sudan and arrived in US in […],*" and "*homeless for about 3 months, has had difficulty finding a shelter*." These entries provided more specific insights into past residences or current housing situations. None of the excerpts included information about the patient’s preference for where they would like to live, such as desired living conditions or ideal locations, or the relevance of their living situation to their personal lives.

#### Personal Interests

Personal interests, capturing details about activities and hobbies that patients did in their spare time or found enjoyable, occurred infrequently (*n* = 24, 2.9% of all instances). Although one entry provided a brief, general reference to activities without much detail (“*Both active”),* the majority (96%, *n* = 23) offered more detailed descriptions of hobbies, outlining specific activities, frequency, or context. Examples include *"He builds remote control drones that he builds for fun," and "In her free time, she enjoys roller-blading and taking care of her grandsons."* Other entries mention “*Enjoys skiing, sailing, biking, reading*," “*enjoys riding motorcycles*,” “*Loves to garden got a raised bed*,” “*attend Mass at church*,” and “Hobby: *watch TV, especially comedy*." None of the excerpts in our data delved into the personal significance of the hobbies and activities.

#### Pets

References to pets were present but also infrequent (*n* = 15, 1.8% of all instances). The majority of pet-related entries (60%, *n* = 9) noted the presence of a pet in the patient’s life without further details. Some examples include "*by herself with 2 cats*" and "*cares for multiple pets.*"

Other entries (40%, *n* = 6) included more specific information about the pets, such as names, ages, or breeds. Examples include "*dog (english lab),*" "*5 yo pit bull*," "*Lives with: dog drew and cat fifi*," and "*Has a German Shepard* (sic)." These descriptions gave a clearer picture of the pets' identities, offering more personalized insights into the patient’s life. None of the excerpts in our sample included explicit information about the significance or emotional importance of the pets to the patient.

#### Travel

References to travel were relatively infrequent (*n* = 5, 0.6% of all instances), focusing primarily on specific locations visited both locally and globally. As previously noted, we did not include entries about travel (or lack of travel) when mentioned solely in the context of describing illness risk (e.g., diarrheal illness) rather than the patient’s lifeworld. Included travel-related entries typically provided information about the specific places the patients had visited. For example, "*travels to NYC a lot*," "*Pt has traveled to Spain, Qatar, Egypt, Argentina, Belfast, Copenhagen*," "*traveled from state to state including Colorado, Minnesota, Illinois and now Baltimore*," and "*He has travelled extensively to Africa and Asia, but only travelled to Ireland in the last 4 years*." These entries focused on the locations themselves. None of the entries explored the personal significance of the places patients had traveled to, nor did they describe the meaning of these experiences in the patients' lives.

#### Goals/Priorities

References to goals and priorities were also a rare category (*n* = 5, 0.6% of all instances), often focusing on the patient’s outlook, resilience, and aspirations for the future. These entries were categorized into two main groups based on the level of detail included. One entry made a succinct and indirect reference to the patient’s goals for their health or life – "*And back driving!*" The remaining excerpts documented broader life goals or priorities that reflected the patient’s personal aspirations and values. Examples include "*She has also been feeling very overwhelmed with legal proceedings after her recent loss, with significant stress, sadness, and weight loss*"; "*she has hope for the future and she wants to get better and spend time with her 6 year old daughter*"; and "*The patient is described as highly independent and high functioning. Her continued independence is of substantial importance to her*." Other entries about goals and priorities mainly centered on emotional well-being, independence, and the pursuit of personal milestones.

### Potentially Stigmatizing Personal Details

Some notes included personal details about challenging life or social circumstances that provided insight into patients’ lifeworlds but may also be perceived as negative or stigmatizing. For example, one note documented that a patient “*holds a sign and occasionally gets jobs or money*,” and another described a patient as “c*urrently living in a tent in the woods*.” Other notes used descriptors such as “*homeless*,” “*on SSI*,” or “*unemployed*,” or referenced strained personal relationships and daily activities, including “*going through a marriage break-up*” and “*spends most of her day at home watching TV*.” Some excerpts also mentioned limited formal education, such as “*completed 10th grade education*” or “s*topped school around 8th grade*.”

### Structured Formats for Personalizing Details

In a subset of notes, physicians recorded personal information using a structured format with repeated labeled fields such as “*Occupation:*,” “*Enjoys:*,” “*Lives with:*,” and “*Marital status:*,” followed by brief patient-specific information, suggesting the use of standardized phrasing or templates incorporated to include personalizing information.

## DISCUSSION

We sought to characterize the prevalence and types of personalizing language—details about patients’ identities, social contexts, life experiences, and perspectives—written by physicians in patients’ medical records. We found that 60% of H&P notes from the inpatient internal medicine units at an urban academic tertiary care hospital contained some personalizing details, while 40% did not, indicating that such documentation was far from universal. Several physicians used similar formats (e.g., “*Occupation:*,” “*Enjoys:*,” “*Lives with:*,” and “*Marital status:*”), suggesting use of note templates to collect personalizing details. Residents were more likely than faculty or fellows to include personalizing language. This is not surprising, since residents are often the inpatient team members charged with taking the most comprehensive history from the patient. Additionally, while residents at our site do not receive standardized or formal curricula on eliciting or documenting personalizing information, they do receive informal, individualized feedback on their history-taking and presentations; cultural norms within the residency and/or guidance from instructors promoting person-centered care may partially explain why a higher proportion of resident notes included personalizing language. Notes for women were less likely to include personal details than those for men. This finding may reflect gender bias in gathering or documenting personalizing information but warrants additional exploration in different settings before firm conclusions are reached, as the absolute difference was relatively small and may have been confounded by other factors we were not able to measure.

The specific dimensions of personalizing language that are included in clinical notes may carry important implications for patient care. While we frequently noted references to family, career, or residence, our results show that domains like personal interests, goals, and priorities were less frequently discussed, despite their potential influence on clinical decision-making and patient experiences.^3,4,19^ Understanding a patient’s priorities can guide shared decision-making, while knowledge of personal interests may foster more meaningful patient-physician connections and recognize patients’ personhoods. The dearth of details about patients’ values and priorities points to missed opportunities for more person-centered care and suggests that many patients may not be adequately characterized as persons within the medical record. More research is needed into which domains best support patient outcomes and how physicians can systematically capture them.

The paucity of details in clinical encounter notes that promote understanding of the patient as a person is important and concerning for two related reasons. First, because personalizing language generally indicates that physicians have elicited information about patients’ lives, its absence may signal missed opportunities to understand patients’ broader contexts. Second, inclusion of personal details in the medical record allows information about the patient’s lifeworld to be shared with the larger care team, and with future physicians, increasing the likelihood of the patient being known as a person and thereby supporting person-centered care.^3,19^ Personalizing details may be particularly helpful in the inpatient hospital setting, where patients are at significant risk of being deprived of their individuality.^20^ They are often unwell, which affects not only their physical health but also their appearance and behavior. They are dressed in hospital gowns instead of their usual clothes, and the hospital environment can be impersonal and removes patients from the contexts of their homes and routines.^21^ Further, admission structures restrict basic aspects of daily life, such as diet and mobility, limiting personal choice and autonomy. Documenting actions or discourse that evoke patients’ lifeworlds may help mitigate this disconnection and the dehumanizing experiences that accompany hospitalization.

Personalization also has the potential to influence physician biases and stereotypes. Robust research has shown that physicians can hold biases against patients, and these biases may be expressed and propagated to other physicians in medical records.^22,23^ In one randomized study, physicians who read a stigmatizing clinical note about a patient with sickle cell disease experiencing a vaso-occlusive crisis had more negative attitudes and recommended less aggressive pain management than those who read a neutral version, demonstrating that clinical note language can shape both attitudes and decisions, and transmit bias from one physician to another.^22^ A recent scoping review found that stigmatizing terms in clinical documentation are common, can reflect underlying racial and other biases, and may negatively affect patients’ experiences and outcomes, underscoring the need to identify and intervene on such language in routine notes.^24^ Addressing these biases and their consequences is necessary for equitable care. Studies have demonstrated that when specific, personalizing information is provided, people are less likely to rely on stereotypical assumptions based on group membership.^5,6^ In this context, personalizing language may counteract stereotypes by providing individuating information.

Personalizing language may also, however, convey or reinforce bias depending on how patients’ lives are described. In our study, personalizing language was largely person-centered, but some instances risked casting patients in a negative light. Personal details about housing instability, financial precarity, disability benefits, unemployment, marital infidelity, or limited formal education can be both clinically relevant and potentially stigmatizing, depending on wording and context. These patterns highlight a central tension: the same kinds of details that can make a patient feel seen may also encode social judgements or negative stereotypes into the record. Thoughtful guidance and training on documenting personalizing information in respectful, non-stigmatizing ways will be essential if efforts to increase personalization are to avoid unintended harms.

In envisioning the future of clinical documentation in the EHR, integrating structured fields to capture personalizing information may enhance the provision of person-centered care. We found that some physicians integrate this information into their existing note templates to systematically elicit details about patients’ lifeworlds, including family, work/education, interests, and perhaps most importantly, goals and priorities. These templated elements are often included in the social history, where they serve to provide a more complete picture of a patient’s social context, extending beyond the typical inclusion of only patients’ health-related habits (smoking, drinking, and drug use). While our study focused solely on personalizing language, this work is conceptually related to research on the social determinants of health, which emphasizes socioeconomic and environmental factors as essential context. Personalizing language is distinguished by representing broader dimensions of individuality, such as priorities, interests, and experiences, enabling physicians to understand their patients as persons, and helping patients feel seen and understood. Any move toward routine or structured personalizing fields, however, should be accompanied by attention to framing, so that system-level changes promote humanizing rather than stigmatizing documentation.

Time is often limited in clinical encounters, and physicians may feel that eliciting and documenting personalizing details competes with clinical tasks. However, exploring a patient’s lifeworld can help build rapport and trust, which are central to effective patient-physician relationships and associated with better medication adherence and clinical outcomes.^25^ Eliciting and documenting personalizing details need not be time-consuming. For example, asking for one”fun fact” or one aspect of life that matters most can surface meaningful information. As clinical documentation increasingly incorporates artificial intelligence, these tools could help capture and integrate personalizing information into notes without adding to physicians’ workloads, though they would ideally be designed to support rather than replace physicians’ engagement with patients' stories.^26–28^ Our findings suggest that such efforts should focus not only on increasing the amount of personal information, but also on helping physicians document it in accurate, non-stigmatizing ways, in alignment with patients’ own priorities.

Our study has several limitations. Our data were limited to notes written in a H&P format from 2017. It is possible that physician documentation practices have changed since then; additional studies using more recent data would be helpful. Second, given our sample was drawn from the internal medicine service of a single urban academic tertiary care hospital serving a predominantly Black patient population with complex medical conditions, patterns observed here may differ from those in other settings or with different patient populations. Third, a small proportion of notes (9%) arose from intensive or critical care settings; whether illness severity and care context influenced whether and how physicians elicit and document personalizing information remains undetermined. Fourth, some histories may have been partly derived from family or other proxies rather than directly from patients, which may have influenced both the amount and nature of personalizing details documented. Lastly, the observed prevalence of personalizing language may reflect physician preferences and perceptions of note purpose, as some physicians may view encounter notes solely as a means of documenting clinical information and may include personal details only when deemed to be directly relevant to diagnosis or management. Understanding how institutional norms, documentation requirements, and local training shape both the presence and tone of personalizing language will be important for designing interventions that promote person-centered, non-stigmatizing notes.

In conclusion, most personalizing language in clinical notes focused on family, career, and residence, while dimensions such as personal interests, support networks, and goals and priorities were limited. Moreover, more than one-third of H&P notes did not contain any personalizing language. The personalizing language documented varied in tone, encompassing both humanizing and potentially stigmatizing descriptions. We recommend further studies to test methods to promote inclusion of personalizing details in the EHR, and to evaluate the impact of personalizing language on physician attitudes, relationships with patients, and quality of care, and the conditions under which such documentation supports or undermines equity and respect for patients.

## Data Availability

The dataset is not publicly available because it contains protected health information (PHI).
